# Perception of and Attitude towards Hepatitis B Infection among Saudi Pregnant Females Attending Antenatal Care Unit in Al-Ahsa City, Kingdom of Saudi Arabia

**DOI:** 10.7759/cureus.6673

**Published:** 2020-01-16

**Authors:** Meshal Al-Essa, Abdulwahab Alyahya, Abdulatif Al Mulhim, Abdulaziz Alyousof, Mohammad Al-mulhim, Abdallah Essa

**Affiliations:** 1 Medicine, College of Medicine, King Faisal University, Al-Ahsa, SAU; 2 Internal Medicine, College of Medicine, King Faisal University, Al-Ahsa, SAU; 3 Gastroenterology - Internal Medicine, College of Medicine, King Faisal University, Al-Ahsa, SAU

**Keywords:** antigen, hbv carriers, vaccine, infection, hepatitis b

## Abstract

Objective

This study aimed to assess the knowledge and attitudes of pregnant females in Al-Ahsa city, Kingdom of Saudi Arabia (KSA) toward hepatitis B virus infection.

Methods

A cross-sectional study was done at the Maternity and Children’s Hospital, Al-Ahsa. A total of 422 of every third pregnant women were recruited from 6/12/2019 to 20/12/2019. Self-administered questionnaire was provided that contained three aspects: sociodemographic, perception and source of information about hepatitis B, and attitude toward hepatitis B infection. Analysis was performed using SPSS version 21 (IBM Corp., Armonk, NY).

Results

A total of 422 pregnant women participated in this study with a response rate of 93.7%. Among them, 44.79% had a university degree or higher education level, about 82% had information about hepatitis B virus (HBV) during their pregnancy, 0.9% knew a person with HBV, 48.1% knew that hepatitis B is caused by virus, 72% knew that hepatitis B has vaccine, 41.9% knew that hepatitis B spreads via mother, 79.6% were willing to do hepatitis B test during pregnancy, 80.1% were willing to allow for kids’ vaccination against HBV, and 83.4% were willing to allow their kids for hepatitis B testing. There was a significant relationship between the level of education and the knowledge score. And there was a significant relationship between the level of education and attitudes score.

Conclusion

There is insufficient knowledge among pregnant women regarding hepatitis B infection, while pregnant women showed remarkably positive attitudes regarding therapy and immunization. So, we highly recommend for awareness campaigns about viral hepatitis regarding means of transmission, and possible treatment options.

## Introduction

Chronic hepatitis B virus (HBV) infections remain a major public health issue worldwide despite availability of effective vaccine and potent antiviral treatments [1]. Chronic hepatitis B virus infection affects approximately 350 million people worldwide, half of whom acquired the infection from perinatal transmission or in early childhood [2].

Perinatal transmission is one of the commonest modes of HBV transmission worldwide. This perinatal transmission of HBV leads to severe long-term sequelae [3]. Children born to mothers who are positive for hepatitis B surface antigen (HBsAg) and hepatitis B e-antigen (HBeAg) have a 70-90% chance of perinatal acquisition of HBV infection, and over 85-90% of them will eventually become chronic carriers of the disease. Chronic carriers of HBV have an increased lifetime risk of dying from hepatocellular carcinoma and liver cirrhosis (25% risk), and remain the main reservoir for continued transmission of HBV [4]. Many of them eventually become mothers themselves, thus perpetuating the cycle [5].

The Global Advisory Group on the Expanded Program on Immunisation recommended that countries with a more than 2% prevalence of HBV carriers should add hepatitis B vaccine into their routine infant immunization schedules, a recommendation which was endorsed by the World Health Assembly [6]. Consequently, the routine screening of pregnant women for HBsAg is recommended by the World Health Organisation [7].

Several studies reported the prevalence of HBV in Saudi Arabia among the general public, school students, blood donors, health care workers, and pregnant women. It has shown that HBV infection in Saudi Arabia is acquired mainly through horizontal and vertical transmission similar to what is observed in other endemic countries. It has been reported that 5-10% of the population were infected with HBV [8-10]. Epidemiological data in pregnant Saudi women indicate about 4% prevalence in this population overall. However, despite a significant decline in the prevalence of HBV infection in Saudi Arabia, the disease continues to cause significant morbidity and mortality and imposes a great burden on the country’s health care system. Compared with other parts of Saudi Arabia, a higher prevalence of HBsAg was found in the eastern region of the country [11].

Pregnant women are vulnerable and if infected can transmit infection to infants, children, health workers during delivery as well as to sexual partners. Major risk factors identified in studies carried out among pregnant women and women of childbearing age include, level of education, history of blood transfusion, surgery, abortions, sexual transmitted infection, higher mean parity, early sexual debut, polygamy and higher numbers of sexual partners [12,13].

So, the level of awareness of the pregnant females about HBV infection plays an important role in HBV prevalence. We suspect that one of the explanations of this high HBV prevalence in eastern region of Saudi Arabia is deficient knowledge on infection with the HBV, especially regarding its prevention in the fertile female population. Hence, this study aimed to assess the knowledge and attitudes of pregnant females in Al-Ahsa city, Kingdom of Saudi Arabia (KSA) toward hepatitis B virus infection.

## Materials and methods

A cross-sectional study was conducted in antenatal care clinics in Al-Ahsa region. The hospital that included was Maternity and Children’s Hospital Al-Ahsa. The study was carried out between 6/12/2019 and 20/12/2019. In this study, 422 pregnant women were included. The sampling technique of this study was systematic sampling of every third pregnant female visitor to the antenatal care clinic of the hospital who was included in the study based upon the two weeks visits to the clinics. Participants were included from four antenatal clinics of Maternity and Children’s Hospital Al-Ahsa based upon the number of data collection. The data were collected in this study by self-administered questionnaire which was adopted from a relevant literature to answer the study objective [14].

The questionnaire was divided into three main parts: the first part was regarding sociodemographic data including age, occupation, education, and marital status, etc., while the second part was about perception and source of information about hepatitis B, and the third part was about attitude toward hepatitis B infection.

Data were stored as database with no access except for those who were authorized. Then the data were coded, entered into Microsoft Excel sheet, and analysis was performed using Statistical Package for Social Sciences (SPSS) version 21 (IBM Corp., Armonk, NY). Descriptive analysis was used for participants’ characteristics, Chi-square was used to compare participants’ attitude of HBV and the independent variables to determine the association, logistic regression was used to estimate the adjusted odd ratio (OR), and inferential statistics were represented by P value (<0.05) and confidence interval (Null hypothesis = 1 for OR).

## Results

**Figure 1:**
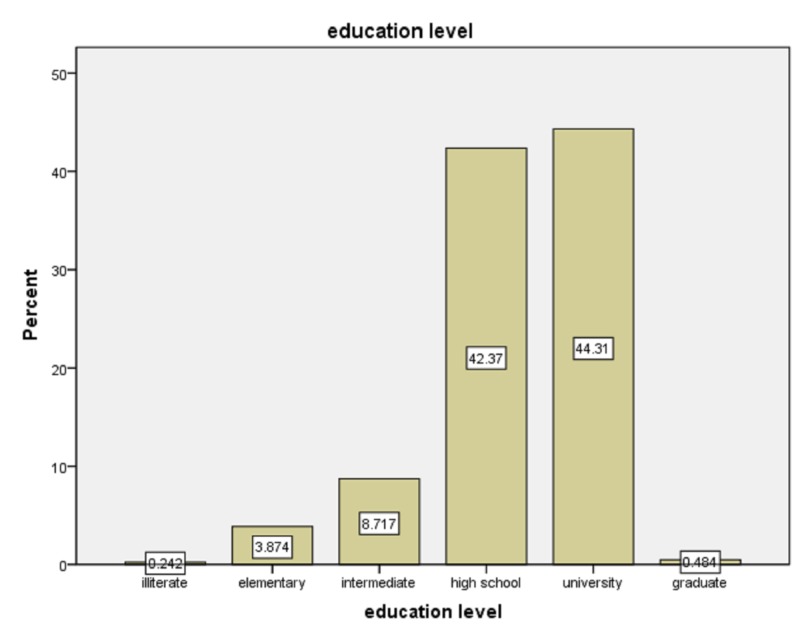
The education level of participants

Of the 450 pregnant women who were approached, 422 agreed to participate with a response rate of 93.7%. The mean age of the participants was 30.89, while the majority of participants’ education levels were high school and university as shown in Figure 1.

Regarding the awareness data, about 82% of participants reported that they received information about HBV during their pregnancy, around 15.9% reported that they did not receive any information about it. Also, 18.5% said they know a person with HBV, while 76.5% said they do not know a person affected with HBV. Moreover, 0.9% reported to having HBV while 95.3% denied having HBV as shown in Table 1.

**Table 1:** Awareness of participants about hepatitis B virus (HBV)

Question	Yes	No	Missing	Total
Q1: Heard about hepatitis B?	82%	15.9%	2.1%	100%
Q2: Know a person has hepatitis B?	18.5%	76.5%	5.0%	100%
Q3: Are you having hepatitis B?	0.9%	95.3%	3.8%	100%

Educational level and awareness

**Figure 2:**
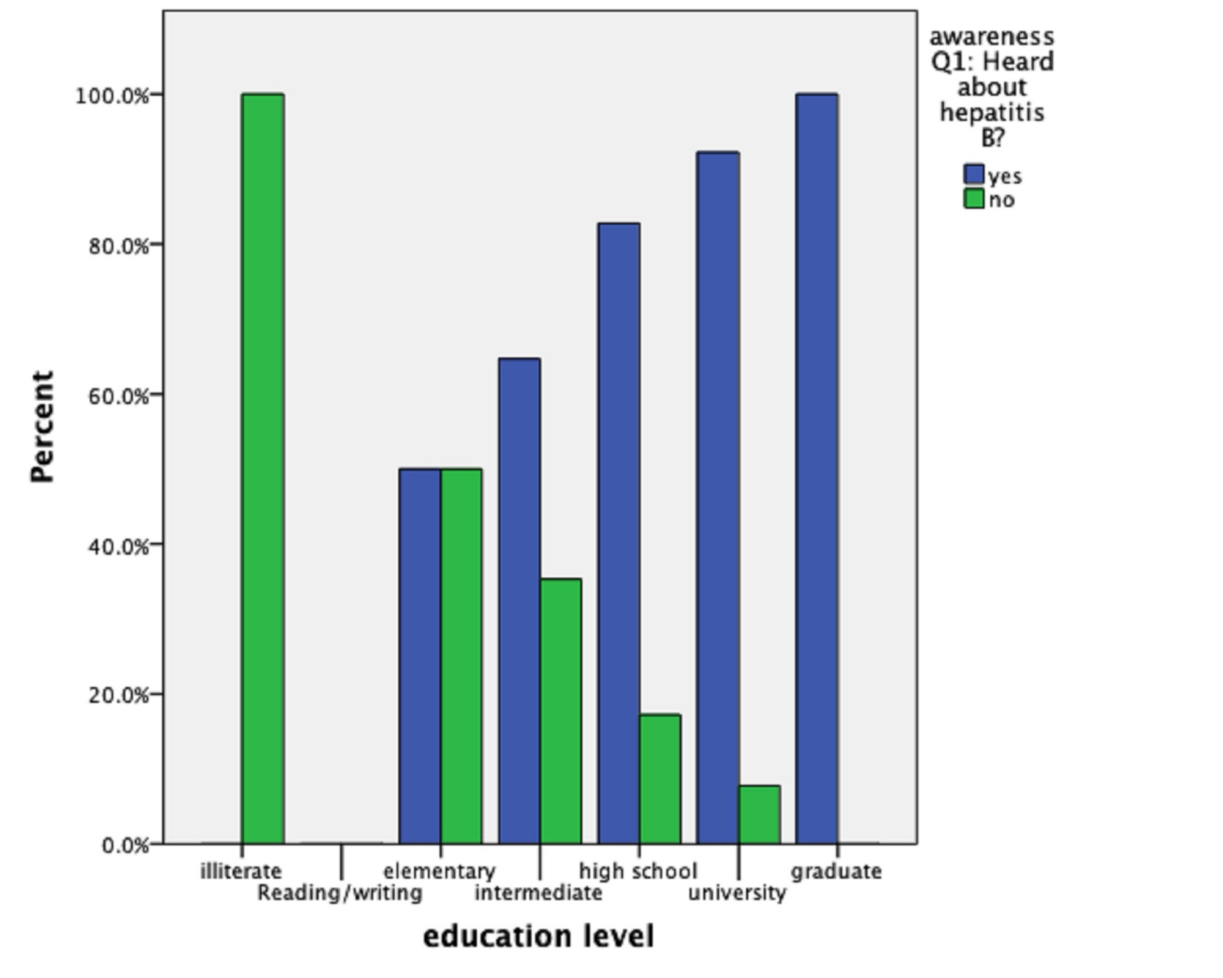
Educational level and awareness. Q1: Heard about hepatitis B?

For Question 1 (Heard about hepatitis B?), one illiterate participant (100%) said ‘no’, eight elementary level participants (50%) said ‘no’ and eight (50%) said ‘yes’ from a total of 16 (100%) participants, 12 intermediate level participants (35.2%) said ‘no’ and 22 (64.8%) said ‘yes’, 144 high school level participants (82.7%) said ‘yes’ from a total of 174 (100%) participants, 166 university level participants (88.2%) said ‘yes’ out of 180 (100%) participants as shown in Figure 2.

Regarding the knowledge data

For Question 1 (Hepatitis B caused by virus?), 203 (48.1%) of the participants said ‘yes’, 27 (6.4%) said ‘no’ and 190 (45%) said ‘I don’t know’ with a total of 420 participants and a missing of two participants. For Question 5 (Hepatitis B has vaccine?), 304 (72%) said ‘yes’, 13 (3.1%) said ‘no’ and 104 (24.6) said ‘I don’t know’ with a total of 421 participants and a missing of one participant. For Question 6 (Hepatitis B spreads via blood?), 200 (47.4%) said ‘yes’, 43 (10.2%) said ‘no’ and 172 (40.8%) said ‘I don’t know’ with a total of 415 participants and a missing of seven participants. For Question 7 (Hepatitis B spreads via sex?), 119 (28.2%) said ‘yes’, 102 (24.2%) said ‘no’ and 198 (46.9%) said ‘I don’t know’ with a total of 419 participants and missing of three participants. For Question 8 (Hepatitis B spreads via mother?), 177 (41.9%) said ‘yes’, 38 (9.0%) said ‘no’ and 206 (48.8%) said ‘I don’t know’ with a total of 421 participants and a missing of one participant. For Question 9 (Hepatitis B spreads via needle?), 187 (44.3%) said ‘yes’, 47 (11.1%) said ‘no’ and 185 (43.8%) said ‘I don’t know’ with a total of 419 (99.3%) participants and a missing of three (0.7) participants as shown in Table 2.

**Table 2:** Knowledge of participants about hepatitis B virus (HBV)

Category		Frequency	%
Q1: Hepatitis B is caused by a virus	Yes	203	48.1%
	No	27	6.4%
	Don’t Know	190	45%
Q2: Hepatitis B infection can lead to liver cancer	Yes	117	27.7%
	No	44	10.4%
	Don’t Know	258	61.1%
Q3: Hepatitis B infection can lead to cirrhosis (scarred liver)	Yes	220	52.1%
	No	17	4.0%
	Don’t Know	170	40.3%
Q4: A person can be infected with hepatitis B and not have any symptoms of the disease	Yes	163	38.6%
	No	77	18.2%
	Don’t Know	181	42.9%
Q5: There is a vaccine for hepatitis B	Yes	304	72%
	No	13	3.1%
	Don’t Know	104	24.6%
Q6: Hepatitis B can be transmitted through blood transfusion	Yes	200	47.4%
	No	43	10.2%
	Don’t Know	172	40.8%
Q7: Hepatitis B can be transmitted through unprotected sexual intercourse	Yes	119	28.2%
	No	102	24.2%
	Don’t Know	198	46.9%
Q8: Hepatitis B can be transmitted from mother to fetus	Yes	177	41.9%
	No	38	9%
	Don’t Know	206	48.8%
Q9: Hepatitis B can be transmitted through use of unsafe needles or sharps	Yes	187	44.3%
	No	47	11.1%
	Don’t Know	185	43.8%
Q10: An individual can be infected by both hepatitis B and HIV	Yes	166	39.3%
	No	31	7.3%
	Don’t Know	224	53.1%

About the attitude data

For Question 1 (Hepatitis B testing during pregnancy), 336 (79.6%) said ‘yes’, 48 (11.4%) said ‘no’ and 34 (8.1%) said ‘I don’t know’ with a total of 418 (99.1%) and a missing of four (0.9%) participants. For Question 2 (Allow kids for vaccine for HBV), 338 (80.1%) said ‘yes’, 40 (9.5%) said ‘no’ and 40 (9.5) said ‘I don’t know’ with a total of 418 (99.1%) and a missing of four (0.9%) participants. For Question 3 (Allow kids for antibodies for hepatitis B), 227 (53.8) said ‘yes’, 108 (25.6%) said ‘no’ and 78 (18.5%) said ‘I don’t know’, with a total of 413 (97.9%) and a missing of nine (2.1%) participants. For Question 4 (Having drugs to prevent HBV), 359 (85.1%) said ‘yes’, 22 (5.2%) said ‘no’ and 35 (8.3%) said ‘I don’t know’ with a total of 416 (98.6%) and a missing of six (1.4%) participants. For Question 5 (Allow kids for hepatitis B testing), 352 (83.4%) said ‘yes’, 28 (6.6%) said ‘no’ and 40 (9.5%) said ‘I don’t know’, with a total of 420 (99.5%) and a missing of two (0.5%) participants as shown in Table 3.

**Table 3:** Attitude of participants toward hepatitis B virus (HBV)

Category		Frequency	%
Q1: Are you willing to be screened for hepatitis B during an antenatal care visit (blood test)?	Yes	336	79.6%
	No	48	11.4%
	Don’t Know	34	8.1%
Q2: Are you willing to let your baby receive HBV vaccine?	Yes	338	80.1%
	No	40	9.5%
	Don’t Know	40	9.5%
Q3: If you got HBV infection, are you willing to let your baby receive anti-HBV antibodies?	Yes	227	55.0%
	No	108	26.2%
	Don’t Know	78	18.9%
Q4: If you got HBV infection, are you willing to take drugs that are known not to harm the developing baby in pregnancy to prevent transmitting HBV to your baby?	Yes	359	85.1%
	No	22	5.2%
	Don’t Know	35	8.3%
Q5: Are you willing to take your baby back to the clinic to test his/her HBV status a few times during the 1st year after birth?	Yes	352	83.4%
	No	28	6.6%
	Don’t Know	40	9.5%

A chi-square test of independence was performed to examine the relation between education level and knowledge about HBV vaccine. There was a statistically significant relationship between these variables, X^2^ (10, N = 412) = 29.619, p = .001. Also, a statistically significant relationship was found between education and some of HBV transmission modes like blood, and needles. Moreover, there was a statistically significant relationship between education and the willing of participants to let their newborn to receive an HBV vaccine of, X^2^ (10, N = 409) = 18.538, p = .047.

## Discussion

This study aimed to measure the knowledge and attitudes of pregnant females in Al-Ahsa city, KSA toward hepatitis B virus infection. As a result, we collected the responses of 422 participants, and recorded and compared demographical, awareness, and health attitudes-related data.

This study revealed that there is a significant relationship between the level of education and the knowledge score, that is to say, the number of questions answered correctly. This correlation was noted when comparing the level of education and attitudes score, i.e., positive attitudes toward vaccination and treatment, where those with higher education level were more likely to answer correctly. This is consistent with the current literature as multiple studies revealed that low health literacy was observed among those with high school or lower levels of education, and among those, the rate of disease and poor health was higher [15,16]. On the flip side, other studies revealed significant relationship between higher levels of education and health literacy, and the likelihood of adopting health promoting activities was higher among those with higher education [17,18]. When comparing the knowledge score of participants to their income, there was a statistical significance. However, this difference was not implicated in the attitudes score. That is to say, even though, there’s a difference in knowledge scores, the attitudes of participants were similar when compared to their income levels. Similarly, there was a difference between age groups regarding knowledge score, but that difference was not seen in attitudes when compared to the age.

Overall, we observed a lack of knowledge regarding viral transmission. For instance, only 47% of participants recognized that HBV is transmitted by blood compared to 85.8% in Vietnam [19], 46% to 75% in China [14,20], and 10% in Nigeria [21]. Only about 28% of the participants were aware of sexual transmission compared to 75% in Vietnam, 46% to 52% in China, and 41% in Nigeria [14,19-21]. Although it is considered routine to test for HBV during the antenatal visits [22], it is necessary to educate pregnant women on the risks of infection and the means of transmission, in addition to emphasizing the benefits of vaccination.

Up to 40% of the cases of HBV are transmitted perinatally, and this particular type of acquisition of the disease is associated with significantly higher infection-related morbidity and mortality [23,24]. Furthermore, the majority of patients with chronic hepatitis B infection, acquired the virus at the perinatal period, unfortunately, more than half of the women questioned (57.8%) are not aware of the possibility of vertical transmission [25].

A 2012 study revealed that treatment with Telbivudine during pregnancy significantly improved disease outcomes regarding fetal transmission, viral load, and liver enzymes normalization [26]. Furthermore, a meta-analysis examining 32 randomized controlled trials investigating the effects of hepatitis immunoglobulin injection in preventing vertical transmission revealed that compared to the controls, those treated with immunoglobulins were 85% less likely to acquire the infection, defined by viral DNA status OR 0.15, 95% CI [0.07, 0.30] [27]. We measured the likelihood of the participants to undergo treatment with antivirals and with hepatitis immunoglobulins had they acquire the infection. We found that 85.1% of the participants (359:416), were likely to take antiviral treatment to prevent infection transmission, provided there are no health risks to their infants.

We found a high rate of positive attitudes regarding vaccination. That is to say, 80.1% of the participants were likely to allow their children’s vaccination. This attitude is similar to that of Vietnam and China, 86% and 89%, respectively [19,28]. This finding could be explained by HBV vaccines being included in the vaccination schedule of children by the ministry of health. The vaccination was included in children immunization schedule in 1989 and was aimed toward infants, preschool and school children, as well as high risk individuals, e.g., those on hemodialysis, which accounted in the significant drop of the incidence of the disease [11,29]. Nevertheless, immunization coverage of pregnant females is still lacking according to a 2008 study that revealed 79% of the pregnant females included were nonimmune [30]. Awareness campaigns on hepatitis B infection, transmission, treatment and vaccination should be undertaken, to lower the burden of the disease, especially as it is preventable. Further studies are needed to measure the trends of prevalence and immunization coverage.

## Conclusions

As speculated, the present study revealed that the degree of knowledge regarding hepatitis B infection is poor among the subjects involved. On the other hand, the participants showed remarkably positive attitudes regarding therapy and immunization. Thus, we highly recommend awareness campaigns on viral hepatitis regarding means of transmission, and possible treatment options, and also emphasizing the importance of vaccination to newborns as well as to nonimmune mothers postpartum.
